# Developing Fall-Impact Protection Pad with 3D Mesh Curved Surface Structure Using 3D Printing Technology

**DOI:** 10.3390/polym11111800

**Published:** 2019-11-01

**Authors:** Jung Hyun Park, Jeong Ran Lee

**Affiliations:** Department of Clothing and Textiles, Pusan National University, Busan 46241, Korea; jhpark1391@pusan.ac.kr

**Keywords:** impact protection pad, 3D mesh, 3D printing, 3D body scan, 3D modeling, force attenuation

## Abstract

In this study, we present the development of fall-impact protection pads for elderly people using three-dimensional (3D) printing technology. To develop fall-impact protection clothing, it is important to maintain the functionality of the protection pad while ensuring that its effectiveness and appearance remain optimal in the process of inserting it. Therefore, this study explores the benefit of exploiting 3D scan data of the human body using 3D printing technology to develop a fall-impact protection pad that is highly suited to the human body shape. The purpose of this study was to present a 3D modeling process for creating curved protective pads comprising a hexagonal mesh with a spacer fabric structure and to verify the impact protection performance by printing curved pads. To this end, we set up a section that includes pads in the 3D human body scan data and extracted body surface information to be applied in the generation of the pad surface. The sheet-shaped hexagonal mesh structure was cut and separated according to the pad outline, and then deformed according to the curved surface of the human body. The pads were printed, and their protection performance was evaluated; a 79.2–81.8% reduction in impact force was observed compared to similar cases in which the pads were not used.

## 1. Introduction

According to a report by the Korea Center for Disease Control and Prevention, an increasing number of elderly people over the age of 65 are being hospitalized due to falls [1]. Furthermore, research indicates that following transport accidents, injuries resulting from falls were the leading cause of hospitalization. Half of the fall patients were hospitalized for more than two weeks, with patients with hip fractures having the longest hospital stays. Falls frequently occur while moving on the road in winter, especially among elderly women.

It is becoming increasingly important to prevent the elderly from falling. Wearing hip protectors can prevent falls or reduce the damage caused by falling [2,3,4,5]. However, existing hip protectors are not suitable for use with daily clothes; they are not widely utilized because of aesthetic limitations. Furthermore, they are not comfortable [6,7]. Therefore, it is necessary to develop an optimized impact protector with due consideration for body characteristics and motion. Additionally, it is necessary to improve the wearing satisfaction by designing the impact protector in a form that is suited to the shape and motion of the human body while maintaining its protection performance.

To develop a fall-impact protection pad that is highly compatible with the human body, utilizing 3D scan data of the human body through 3D printing technology is advantageous [8]. Three-dimensional printing is a manufacturing technology that prints materials layer by layer based on 3D modeling information. This printing technology is widely used in small-scale production and prototype development, because it can produce 3D objects relatively easily and efficiently [9,10,11]. It is already in use in the fields of machinery, aerospace, automobiles, consumer goods/appliances, and medicine/dentistry [12,13,14,15,16,17,18,19]. The development of 3D computer-aided design (CAD), the emergence of low-cost, personal 3D printers, and the cost reduction of printing materials have fueled the increasing popularity of 3D printers [20,21,22].

In the fashion field, 3D printed clothing serves mainly artistic purposes; i.e., it is generally not functional. Such technology is actively deployed in producing accessories, such as hats, shoes, bags, and glasses, because they are small in size and require design versatility, as their production involves a wide range of materials and designs [23,24]. Valta and Sun [25] demonstrated the possibility of introducing 3D printed parts of a garment using the fused deposition modelling (FDM) technique. Eom et al. [26] studied the thermal transmission properties of 3D printed materials with a 3D spacer fabric structures for use in cold-weather clothing. Pei et al. [27] investigated the adhesion of polymer materials printed directly onto fabrics using entry-level FDM machines. Sanatgar et al. [28] analyzed 3D printing process parameters such as extruder temperature, construction platform temperature, and printing speed on the adhesion of polymers and nanocomposites to fabric. Although attempts have been made to apply 3D printing technology to various structures and forms in the field of textiles, it is still in the early stages of development [29].

Three-dimensional printing technology can be applied to develop a protective pad with a complex three-dimensional structure to fit the curved shape of the human body. Moreover, the 3D printing method makes it possible to produce prototype products relatively easily with low manufacturing costs compared to the use of conventional foam materials made by injection molding.

Therefore, in this study, we propose the design of a curved protection pad based on human body surface information using 3D printing technology that, in addition to being able to produce three-dimensional shapes, has a wide range of shape and structural designs. We further printed out the protection pad and verified the impact protection performance. We extracted the pad surface from the 3D human body scan data of elderly women and applied the hexagonal mesh structure with light and flexible characteristics to the pad shape to develop a pad that provides greater satisfaction in terms of impact protection and wearing comfort. The findings of this study can further reveal the potential of 3D printing technology in the field of functional clothing.

## 2. Experimental Methods

### 2.1. 3D Modeling

Three-dimensional modeling methods can be divided into polygon, non-uniform rational basis spline (NURBS), and solid modeling. The polygon method is based on triangles and a three-dimensional mesh structure. Although it offers fast and light data calculation, its accuracy is relatively poor. The NURBS method uses lines, surfaces, and solids to create three-dimensional shapes; although it requires more computation power than the polygon method, it enables accurate modeling. The solid method can calculate the weight of the actual material, thus making it possible to perform dynamic simulations and achieve modeling that incorporates the properties of the material. It can be used with airplanes, ships, and architectural designs. The calculation cost of the solid method is the highest. In this study, we use the NURBS method, specifically, Rhinoceros 5.0 (Robert McNeel & Associates, Seattle, WA, USA), i.e., the software of the NURBS method widely used in art, design, and architecture. By enabling accurate numerical modeling and polygon mesh editing, it makes it easy to apply 3D human body scan data to surface pad modeling. Therefore, we used it to model the curved surface pad. The pad designed in this study has the following characteristics:

- Curved surface pad fitted to 3D human shape.
- A pad outline with shape properly outlining the impact protection area.
- A hexagonal three-dimensional unit that is closely connected in honeycomb form, maintaining flexibility due to the connection of each structural unit by thin plate.
- A hexagonal, three-dimensional unit structure composed of a surface layer and a spacer layer with an impact-absorbing property similar to that of the spacer fabric.

To design a protection pad with the above characteristics, a 3D modeling process was performed as follows:

- Setting a baseline over the human 3D body scan data (base surface).
- Creating a pad outline, and using it to create a flat surface.
- Placing a pad outline along the body surface, and using it to create a curved surface (target surface) that reflects the body shape.
- Designing a hexagonal mesh structure and extracting a flat pad shape conforming to the pad outline (flat pad with structural information).
- Completing the curved surface protective pad by transforming the pad of the flat hexagonal mesh structure (flat pad with structural information) according to the human body’s curved surface contours (target curved surface) through the base curved surface.

### 2.2. 3D Printing of Protective Pads

In this study, a 3D model of a protective pad was printed using fused deposition modeling (FDM), the most popular 3D printing method. This method is cost- and time-effective. The Cubic on Single 3D printer (CUBICON Inc., Seongnam-si, Korea), that can use flexible filament material, was used in this study, because it is necessary for the protection to have flexible characteristics. The maximum size that can be printed using the Cubicon Single is 240 × 190 × 200 mm (W × D × H); acrylonitrile butadiene styrene copolymer (ABS), polylactic acid (PLA), and thermoplastic polyurethane (TPU) filament with a diameter of 1.75 mm can be used. The printer nozzle size is 0.4 mm. NinjaFlex^®^ (NinjaTek, Manheim, PA, USA). A flexible TPU filament was used for printing the pad of the three-dimensional mesh structure. The melting point of NinjaFlex^®^ is 216 °C; it enables 660% elongation and has 85 Shore A hardness. The 3D model, saved as a stereolithography (STL) file, was sliced using the Cubicreator 2.5.2 (CUBICON Inc., Seongnam-si, Korea) software, and the G-code was created through setting adjustments. The nozzle temperature was set to 230 °C, and the bed and chamber temperature to 40 °C. The thickness of the layer was 0.2 mm, and the printing speed was set to 40 mm/s. Because the protective pads designed in this study were three-dimensional curved surfaces, supporters were created to enable printing. The printing was processed through the temperature setting, filament loading, leveling, extrusion, and layering. After printing, the various part of the pad, from which the supporter was removed, were placed in stretch jersey fabric in accordance with the design, and were combined into a piece of pad by stitching.

### 2.3. Evaluation of Impact Protection Performance

**Figure 1:**
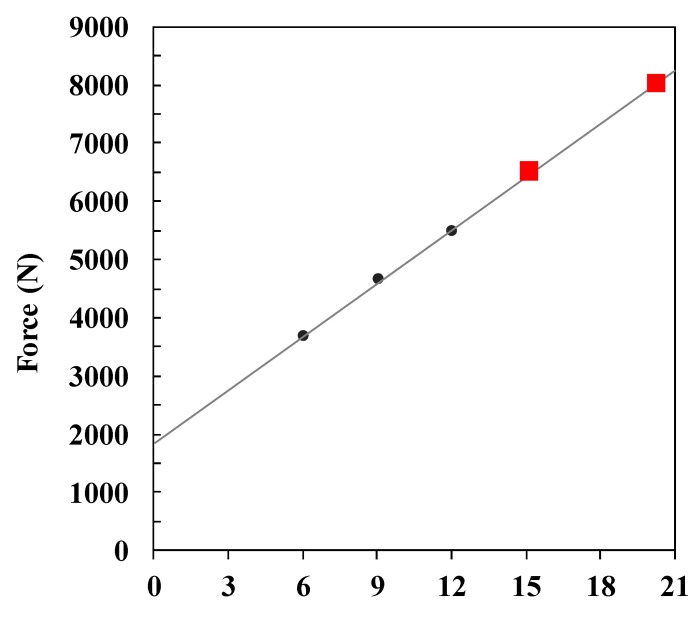
Impact forces measured without pad.

To evaluate the impact protection performance of the pad, a six-pound bowling ball was dropped from varying heights to generate a force in the direction of the ground simulating a situation where the human body is impacted when it falls down. The force generated by dropping the bowling ball from heights of 6 cm, 9 cm, and 12 cm without the pad were measured. Due to the limitation of the equipment, the force at 15 cm and above could not be measured; thus, extrapolation was applied to estimate the value of the force using the results for 12 cm or below. The force generated in the ground direction can be estimated to be ~8000 N at a drop height of 20 cm and ~6500 N at a drop height of 15 cm, as shown in Figure 1.

**Figure 2:**
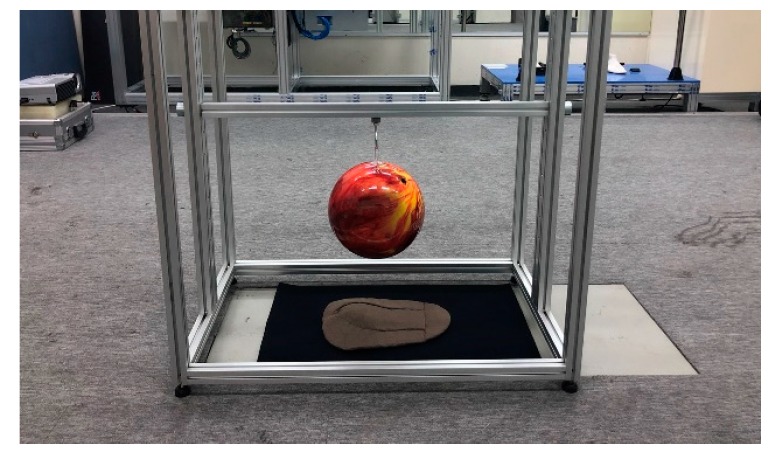
Picture of the impact performance test equipment.

To verify the impact protection of the 3D printing pads, a six-pound bowling ball was dropped from heights of 15 cm and 20 cm onto the force plate on which the pad was placed, and the impact absorption rate of the pad was obtained by comparing the impact value with the situation with no pad (Figure 2). The drop test was performed 10 times at each height. To prevent damage to the sensor and force plate, a 6.0 mm-thick neoprene fabric was laid on the force plate.

### 2.4. Evaluation of Flexural Stiffness

**Figure 3:**
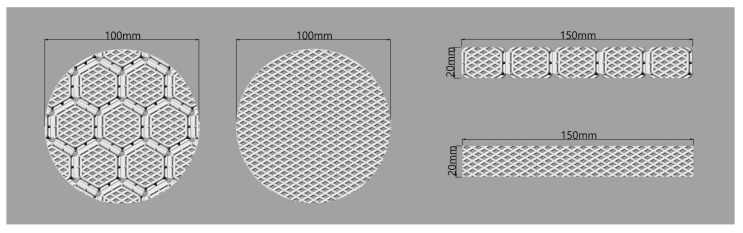
Specimens for flexural stiffness test.

To evaluate the flexural stiffness of 3D printing pads, standard test methods of KS K 0350 (ball bursting method) were used to determine the displacement distance (mm) when constant forces (5 N, 15 N, and 30 N) were applied by a ball bursting tester (Instron, Norwood, MA, USA) on the circular type of 3D printing pads. The diameter of test specimens was 10 cm with a thickness of 1 cm (Figure 3). A greater displacement distance indicates more flexible materials. The strap type of the 3D printing pads was also prepared to measure the flexural stiffness of the 3D printing pads using the standard test methods of KS K 0642 (flexometer method). The size of test specimens was 2.0 cm × 15.0 cm × 1.0 cm (Figure 3). First, the test specimen was placed on the flat region of a flexometer. Then, the test specimen was pushed to a slope region, i.e., 41.5°, as measured using a flexometer. The distance of the test specimen was measured when the end-tip of the test specimen touched the slope region of the flexometer. A greater length of measured distance indicates higher flexural stiffness materials. The flexural stiffness was measured three times and the average value was obtained.

### 2.5. Evaluation of Elongation Rate

The elongation rate (%) was measured with standard test methods of KS K 0521 (strip method) by a universal test machine (Instron 5582, Instron, Norwood, MA, USA). The constant forces (5 N and 15 N) were applied to measure the elongation of the 3D printed pads. The sample dimensions were set to 2.0 cm × 15.0 cm × 1.0 cm (Figure 3). The strain rate was 100 mm/min. The elongation was measured three times and the average value was obtained.

## 3. Results and Discussion

### 3.1. Processing of Body Scan Data

**Figure 4:**
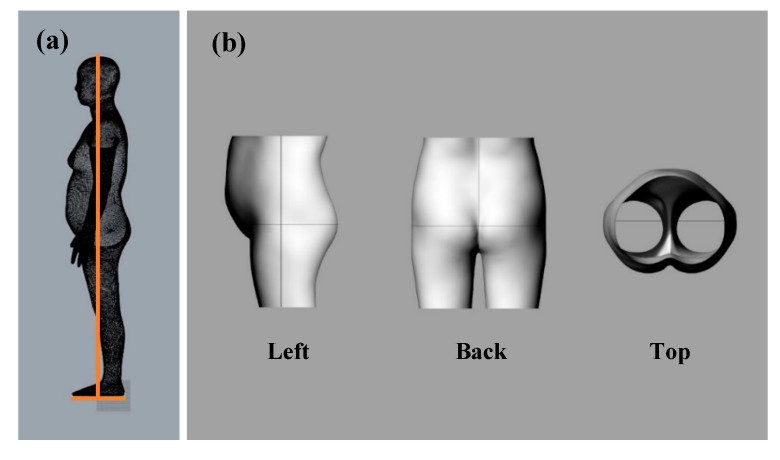
Procedure of body baseline setting (**a**) and human body scan data processing (**b**).

To design a protective pad that fits the human body shape, the human body scan data of women in their 60 s was required. In this study, 3D scan data in the OBJ format, an anthropometric data of Size Korea, was used. The hip circumference, lateral line, and posterior center line were drawn on the human body scan data. To make the modeling process more convenient, the human body was divided into horizontal planes 6.5 cm above the waist circumference and 21.5 cm below the hip circumference; only the hip part was used, as shown in Figure 4.

### 3.2. Creation of Pad Outline and Base Surface

**Figure 5:**
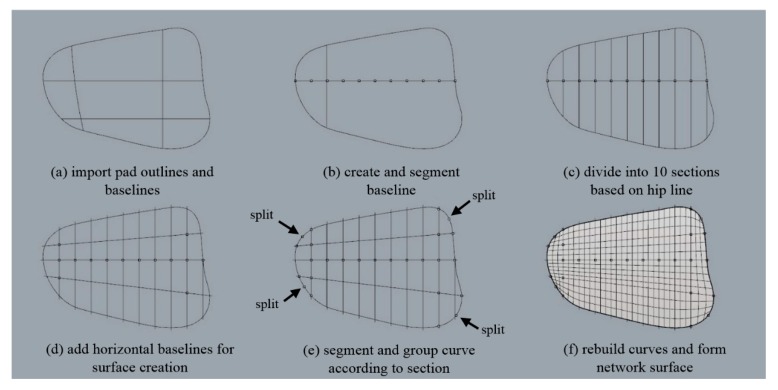
Steps of creating pad outline and base surface: (**a**) import pad outlines and baselines; (**b**) create and segment baseline; (**c**) divide into 10 sections based on hip line; (**d**) add horizontal baselines for surface creation; (**e**) segment and group curve according to section; and (**f**) rebuild curves and form network surface.

In this study, the round pad outline designed using pattern CAD (YUKA) was imported into Rhinoceros 5.0. A sideline was modified by creating a vertical line at the intersection with the hip circumference. As a preprocessing step to modifying the pad outline on a flat surface to fit the 3D human body shape, the curved outline located on the plane was divided. The hip line was divided into 10 parts, following which a vertical line was created at the split point and extended to fit the outline. To form the surface, horizontal baselines were added above and below the hipline. To form a surface using the curved outlines, dividing the outline into the top, bottom, left, and right proved to be effective. Therefore, the outline was split at the point corresponding to the corner. The horizontal baseline was also divided into sections. The elements of the sections were grouped, making it possible to bundle and move them in each section. We created a network surface using the outlines and split lines, and set it as the ‘base surface’ (Figure 5).

### 3.3. Formation of Pad Surface on the Body Surface

**Figure 6:**
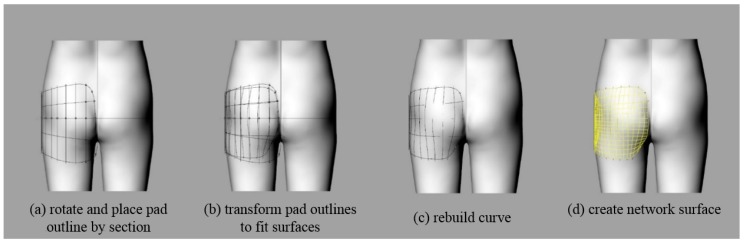
Steps of creating pad outline and base surface: (**a**) rotate and place pad outline by section; (**b**) transform pad outlines to fit surfaces; (**c**) rebuild curve; and (**d**) create network surface.

The outline of the pad was aligned with the baseline (lateral line, hip line) of the human body and rotated for each divided section to approximate the contours of the human body curved surface. The lines constituting the pad surface were drawn over the human body surface and deformed to fit the curved surface shape. Because the 3D body surface was composed of small, triangular meshes, the outlines along the surface were uneven complex lines; therefore, the curves were regenerated to simplify and smooth them. The regenerated curves were used to create the network surfaces that corresponded to the planar network surfaces. The curved network surface was to be used as a ‘target surface’ (Figure 6).

### 3.4. Formation of Three-Dimensional Mesh Structure Planar Pad

**Figure 7:**
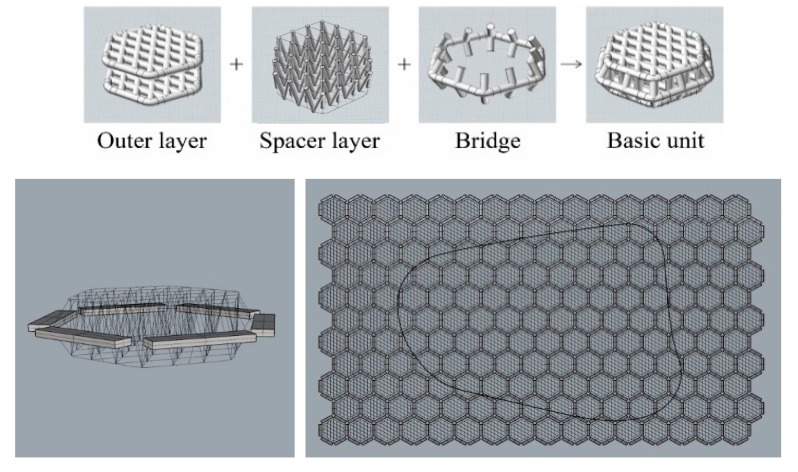
Wireframe of hexagonal mesh structures.

**Figure 8:**
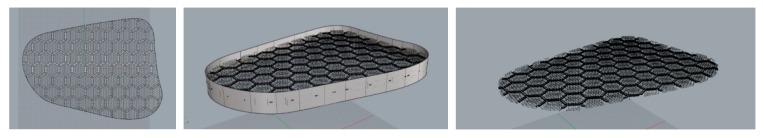
Cutting procedure of the pad structure to match pad shape.

The pad structures were generated using the three-dimensional mesh structure. The diamond-shaped surface layer and the V-shaped spacer layer generated a basic unit, and the units were connected by connectors. The hexagonal structure of the basic unit is densely connected between individual units without empty space. The hexagonal mesh structure can be also flexibly deformed to various directions compared to the rectangular structure. Therefore, due to the connector, the pad moves flexibly with the movement of the human body to improve the wearing comfort. To apply a three-dimensional mesh structure to a curved pad, it is necessary to create a plane by repeating each unit structure, and cutting the repeating structure according to the outline shape. Because it is easier and more efficient to manipulate the curves compared to surfaces or solids, the unit structure forms a wireframe with curves. After repeating the unit structure, as shown in Figure 7, only the components corresponding to the pads were retained by the pad outline surface; the unnecessary parts were deleted, as shown in Figure 8.

**Figure 9:**
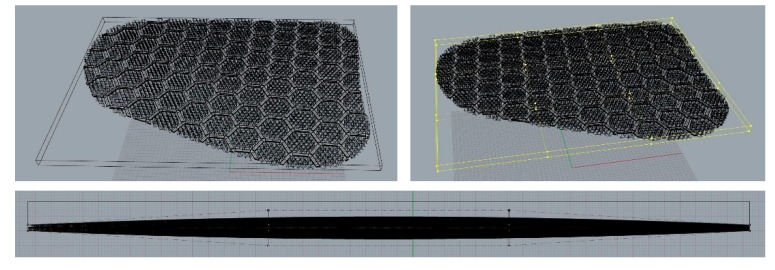
Adjusting pad thickness.

The thickness of the pads was adjusted using the cage function. To achieve a natural appearance when they were inserted into the garment, the pads should be thinner at the edges. Using the cage-edit function on the curves, the thickest part was set to 10 mm, and the thinnest to 4 mm, considering appearance and protection performance, as shown in Figure 9.

**Figure 10:**
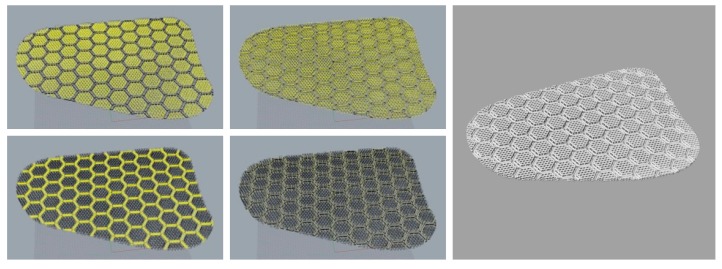
Grouping each pad component.

After the overall wireframe of the pad was complete, the pipe function was applied to the curve to create the surface. It was necessary to group each component, because the pipe sizes of each component varied. The spacer layer had pipes with a diameter of 1.0 mm, and the surface layer and bridge had pipes with diameter of 2.0 mm. The pad on which the pipe was formed is as shown in Figure 10.

### 3.5. Formation of Curved Pad of Three-Dimensional Mesh Structure

**Figure 11:**
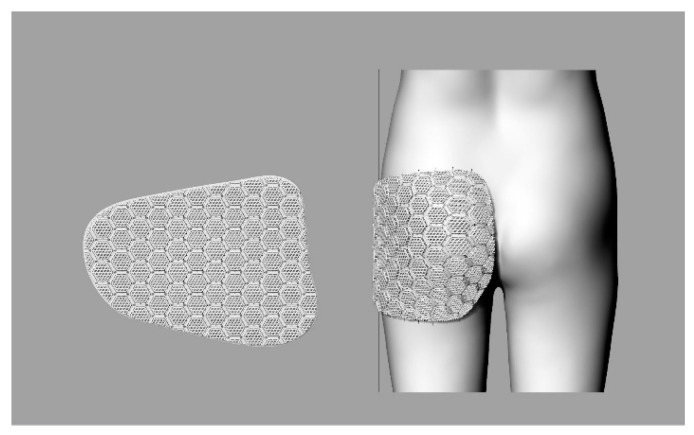
Formation of curved pad of three-dimensional mesh structure.

The curved three-dimensional mesh structure was generated through the following steps. Through the ‘base surface’, formed as described in Section 3.2, the three-dimensional mesh structure was deformed to flow along the ‘target surface’, formed as described in Section 3.3 and shown in Figure 11.

### 3.6. Dividing Pad

**Figure 12:**
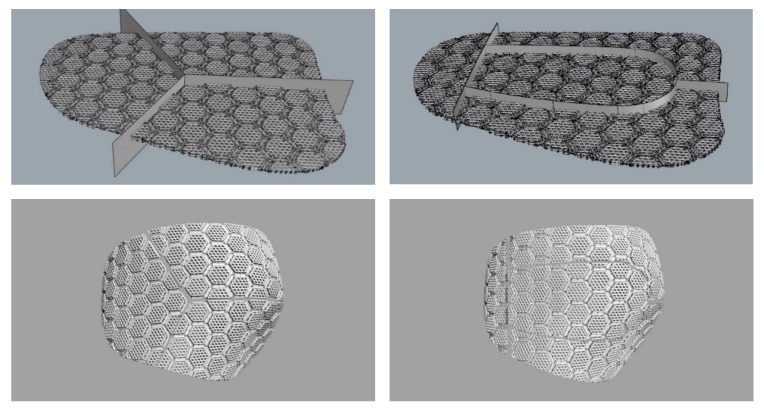
Pad-dividing procedure in the radial split method (**left**) and the elliptical split method (**right**).

The pad was divided, because the size of the curved pad exceeded the printable range. The radial split method for dividing pieces into three by 120° from the center point of the pad, and the elliptical split method for dividing pieces into four according to the sideline and curved surface were used. The elliptical split method was used in this study because, as shown in Figure 12, a comparison of the two types of printed pads revealed that the elliptical split method reflected the curved shape well.

### 3.7. 3D Printing of Final Product

**Figure 13:**
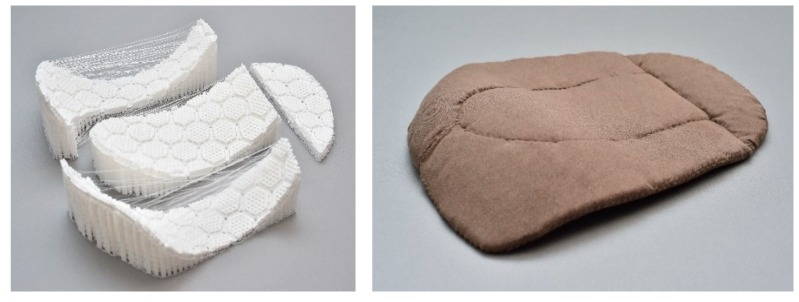
Final product of 3D printing of human body shape.

Figure 13 shows the curved fall-protection pads that fit the 3D human body shape obtained through 3D printing and the fusion of all the pads. After the supporters were removed from the printed four-piece pads, each component was placed between two stretchable fabric layers and fixed as an inseparable pad by sewing the top and bottom fabrics with running stitch and blind stitch.

### 3.8. Impact Protection Performance

**Figure 14:**
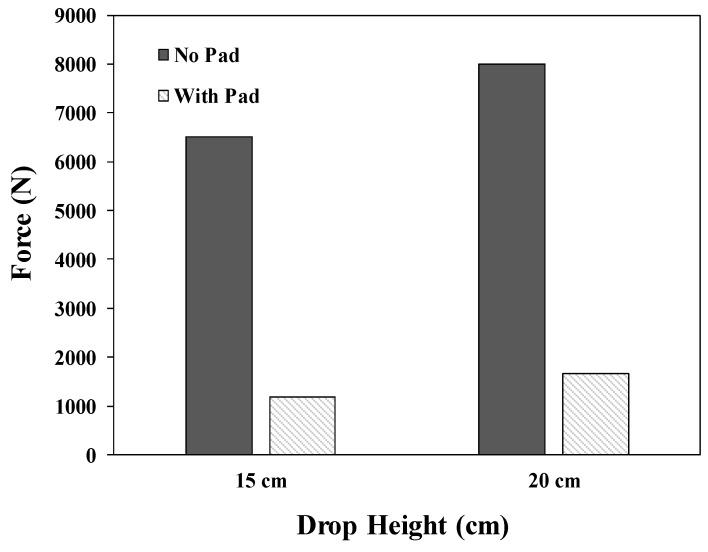
Impact protection performance test with the curved three-dimensional mesh protection pad and with no pad from a height of 15 and 20 cm, respectively.

To verify the impact protection performance of the curved three-dimensional mesh protection pad, an estimated impact force of 6500 N was applied to the pad at a height of 15 cm in the vertical direction. As shown in Figure 14, the average peak value of the vertical force measured on the force plate over 10 repeated tests was 1182 ± 28 N. From a height of 20 cm, an impact force of approximately 8000 N was applied to the pad; the average maximum impact value measured was 1663 ± 78 N.

**Figure 15:**
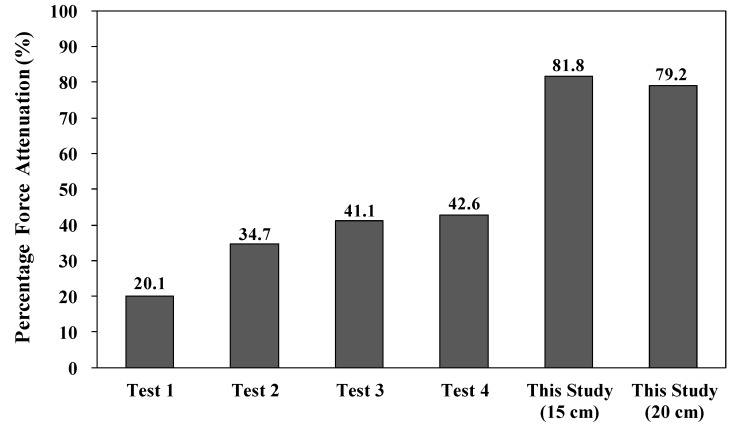
Percentage force attenuations. Tests 1 and 2 were conducted with a polyurethane hip protector on concrete and wooden floors, respectively; Tests 3 and 4 were conducted with a polystyrene hip protector on concreate and wooden floors, respectively [30].

The percentage of force attenuation (%) was calculated to obtain the impact reduction performance of the curved three-dimensional mesh protection pad using the following Equation (1).

(1) $$\mathrm{Percentage}\;\mathrm{Force}\;\mathrm{Attenuation}\;\left(\%\right)=\left(\;1-\;\frac{F_{T}}{F_{0}}\;\right)\;\times 100$$

where *F_T_* is the impact force (N) using the protection pad and *F*_0_ is the impact force (N) without it, respectively. We achieved percentage force attenuations (%) of 81.8 and 79.2 with drop heights of 15 cm and 20 cm, respectively. As shown in Figure 15, Tsushima et al. reported a range of 20.1 to 42.6 using conventional hip protectors fabricated using, e.g., polyurethane and polystyrene elastomer [30]. The percentages of the force attenuations (%) for Tsushima et al. were reproduced based on the impact force from various floor materials without a hip protector. Thus, the results of the percentage force attenuation indicated that the curved three-dimensional mesh protection pad provided excellent impact protection performance.

### 3.9. Flexural Stiffness

**Figure 16:**
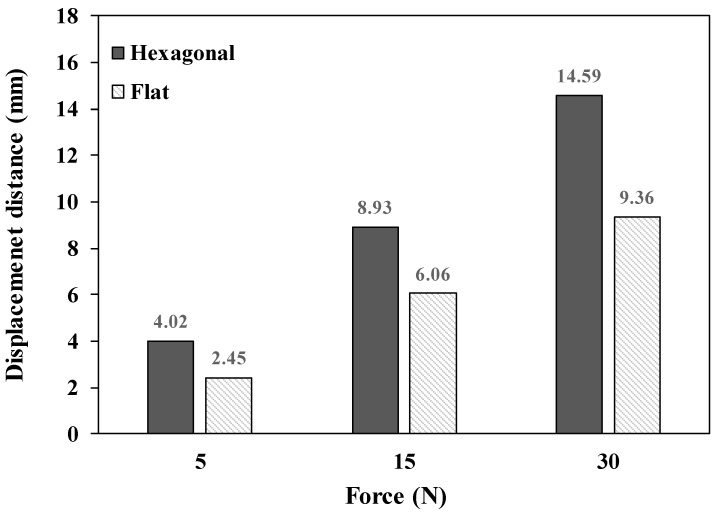
The flexural stiffness tests of 3D printing pads with hexagonal mesh and flat mesh structures, with applied forces of 5 N, 15 N, and 30 N, respectively.

The bending stiffness tests of the circular pads with hexagonal mesh structures and flat mesh structures were performed to investigate the structural effect on the pads. As shown in Figure 16, the displacement distance of the hexagonal mesh structure was 4.02 mm with an applied force of 5 N, 8.94 mm with an applied force of 15 N, and 14.59 mm with an applied force of 30 N, respectively, which was greater than those of the flat mesh structure. By flexometer analysis, the test specimens were pushed to the slope region of the flexometer. The measured distances of the hexagonal mesh and flat mesh structures were 67 mm and 140 mm, respectively. Thus, the hexagonal mesh structure showed higher flexibility than the flat mesh structure in both the circular and linear shape specimens. Therefore, the pad with the hexagonal mesh structure is expected be better suited to the movement of the human body, due to its excellent flexibility.

### 3.10. Elongation Rate

**Figure 17:**
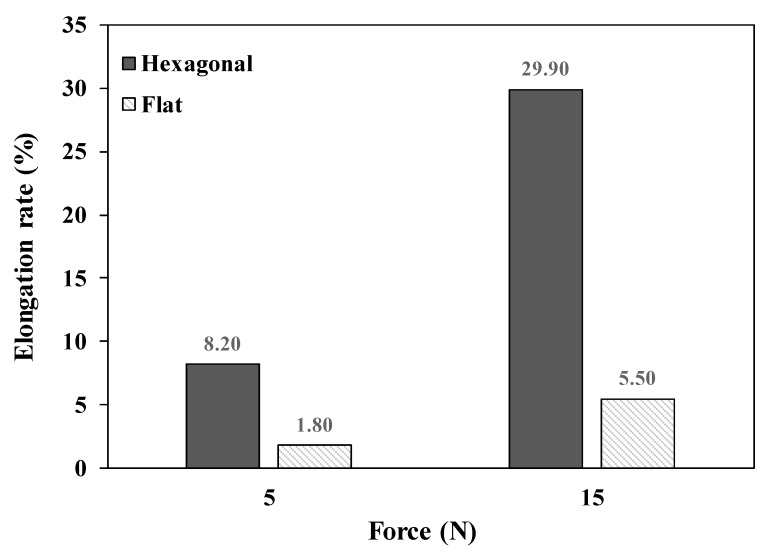
The elongation rates of the hexagonal mesh and flat mesh structures with applied forces of 5 N and 15 N, respectively.

The elongation rate (%) was also measured with the hexagonal mesh and flat mesh structures. As shown in Figure 17, the elongation rates of the hexagonal mesh structure were 8.20% and 29.90% with applied forces of 5 N and 15 N, respectively. In the case of the flat mesh structure, the elongation rates were 1.80% and 5.50% with applied forces of 5 N and 15 N, respectively. Since a greater elongation rate indicates a more stretchable pad, the pad with the hexagonal mesh structure showed 4.6- and 5.4-times higher elongation rates than the pad with the flat mesh structure. Therefore, the hexagonal mesh structure has better stretchability than the flat mesh structure.

## 4. Conclusions

In this study, using 3D printing technology, we designed a new three-dimensional mesh pad, and developed a fall protection pad that fits the human body structure and which yields satisfactory impact protection performance by utilizing the 3D human body scan data of elderly women. When an impact force in the range that can occur in daily life was applied to the protective pad, it was reduced by 79.2 % or more. The uniformly repeated hexagonal mesh units presented in this study are structurally flexible, because they are connected by thin bridges, and the curved protective pads reflect the three-dimensional body shape, making them suitable for daily use. Moreover, the modeling was dynamic to develop impact protection protectors reflecting the body shape. Through several iterative experiments, we developed a reasonable and delicate modeling method that yielded results which are applicable to research on clothing and other fields. Existing 3D printing technology has been deployed to produce very hard products; however, in this study, the printing conditions were finely set, in consideration of the complex characteristics of flexible filament materials. Finally, we believe that our findings foreground the possibility of using 3D printing technology to print complex and elaborate shapes in the field of functional clothing.
